# Phages infecting *Clostridium sporogenes*

**DOI:** 10.1128/mra.00859-25

**Published:** 2025-11-12

**Authors:** Joanna P. Steczynska, Kaleb W. Jackson, Kelly P. Williams

**Affiliations:** 1 Systems Biology Department, Sandia National Laboratories 111651, Livermore, California, USA; Loyola University Chicago, Chicago, Illinois, USA

**Keywords:** phage, *C. sporogenes*, *Clostridium*

## Abstract

*Clostridium sporogenes* is a spore-forming anaerobe found ubiquitously in the environment associated with food spoilage. It is also a gut commensal. We report isolation of phages able to infect *C. sporogenes*, with 30 unique genomes falling into three congeneric species.

## ANNOUNCEMENT

*Clostridium sporogenes* is a common human gut commensal and is associated with food spoilage. It is also a non-toxigenic model organism for *Clostridium botulinum* (1). Here, we report the isolation of 30 unique phages for this organism, whose genomes have been sequenced and annotated. They serve as a useful resource for the development of tools to control *C. sporogenes* and related species.

Multiple sewage samples were harvested from the Livermore Water Reclamation Plant (Livermore, CA, USA) wastewater facility on September 20, 2024. The supernatants were filtered (0.45 mm pore size) and enriched immediately with *C. sporogenes* ATCC 15579. Growth was carried out under anaerobic conditions at 37°C (Don Whitley, A35 chamber, 5% H_2_) in Reinforced Clostridial Medium (Sigma-Aldrich). Resulting plaques were medium to large with clear centers and hazy, irregular edges, except for CS19, which exhibited large, very hazy plaques. Forty-three plaques were purified three times, and genomic DNA was extracted from high titer stocks (>10^8^ pfu/mL; Phage DNA Isolation kit, Norgen Biotek). Libraries were prepared using the Illumina DNA Prep kit and sequenced using the MiSeq V3 150-cycle kit in paired-end mode (Illumina).

**Fig 1:**
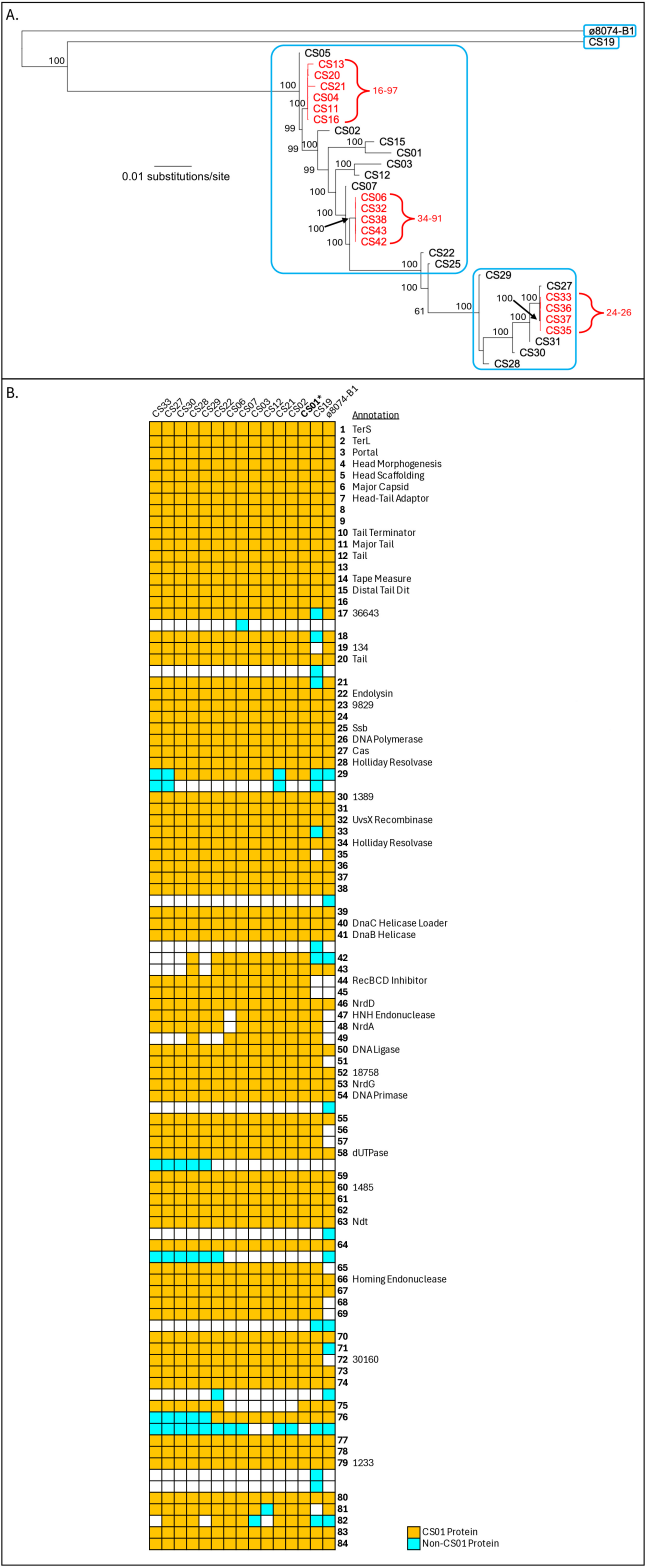
*Clostridium sporogenes* phage comparisons. (**A**) Phylogenetic analysis of 30 new phages plus phi8074-B1. A 54,380-column alignment was prepared for all whole-genome nucleotide sequences using MAFFT v7.526 (with flag --auto)(7) and used to generate an unrooted (shown here as midpoint-rooted) tree (RAxML-NG v. 1.2.2 with flags --tree pars{25},rand{25} --model GTR + G, with 1000 bootstraps)(8). Branch bootstrap support values are given, except for the tight clades in red, where it was not possible to legibly show the branching pattern nor therefore the individual support values; for these, we give the range of support values for the clade. Blue boxes: congeneric species clusters assigned by VIRIDIC. (**B**) Phage proteins were clustered as connected components in all-vs-all BLASTP with a cutoff of 60 bits. Unique orders of these clusters were aligned; those represented in CS01 are colored orange and numbered by the CS01 gene number, while others are colored cyan. Protein labels are derived from Pharokka annotations; numerical labels are PHROG IDs that did not assign function.

Reads were obtained for 42 phages and assembled using SPAdes v3.15.5 (2). Each assembly yielded a single large (~38 kbp) high-coverage contig. Circularization of these contigs was performed by manually trimming one of the ~55 bp terminal repeats typically left for DNA circles by SPAdes, and circularity of the product was verified using ReadStepper with the raw reads (3). Some of the 42 resulting genome sequences were identical, comprising 30 unique sequences (Table 1; Fig. 1A). The genomes all had close (with hits ≥8,000 bits, percent identity ranging from 91.6 to 100, and length from 4,433 to 48,556) BLASTN relationships with each other and with a GenBank entry (NC_019924.1) for another *C. sporogenes* phage, phi8074-B1, but showed no hits to any other viral GenBank entries nor any of the 8630 reference prokaryotic virus genomes listed in the International Committee on Taxonomy of Viruses (ICTV) document MSL40.v1 (4). Phylogenetic analysis is shown in Fig. 1A. Pharokka (5) v1.7.5 (flags: -g prodigal --dnapler) was used for genome annotation, yielding no tRNA genes. Gene annotations (numerous tail genes, lack of integrase or repressor genes) indicated a virulent tailed phage (Fig. 1B), that is, the taxonomic class *Caudoviricetes*. No lower taxonomic ranks could be assigned due to the failure to match any ICTV reference genomes. However, the sequences for the 30 new genomes and for phi8074-B1 were submitted to the VIRIDIC website (6), which placed them into a single genus cluster and four species clusters (one containing only phi8074-B1) (Table 1; Fig. 1A). Default parameters were used for all software unless otherwise specified.

**TABLE 1:** Characteristics of phage genome sequences

Phage	Size (bp)	Coverage	GC content (%)	Protein genes	Species cluster	GenBank accession	BioSample accession
CS01	48329	127.6	35.9	83	1	PV788061	SAMN48543954
CS02	48497	201.3	35.9	83	1	PV788062	SAMN48543955
CS03	48617	145.8	35.9	82	1	PV788063	SAMN48543956
CS04	48496	139.9	36.0	83	1	PV788064	SAMN48543957
CS05	48492	80.9	36.0	83	1	PV788065	SAMN48543958
CS06	47703	140.5	35.8	81	1	PV788066	SAMN48543959
CS07	48501	111.1	35.9	84	1	PV788067	SAMN48543960
CS11	48496	118.4	36.0	83	1	PV788068	SAMN48543961
CS12	48241	81.7	35.9	81	1	PV788069	SAMN48543962
CS13	48511	88.7	35.9	83	1	PV788070	SAMN48543963
CS15	48438	120.6	35.9	83	1	PV788071	SAMN48543964
CS16	48495	106.9	36.0	83	1	PV788072	SAMN48543965
CS19	48481	123.2	35.5	85	3	PV788073	SAMN48543966
CS20	48496	88.2	35.9	83	1	PV788074	SAMN48543967
CS21	48731	86.5	35.9	84	1	PV788075	SAMN48543968
CS22	48378	65.6	35.9	85	1	PV788076	SAMN48543969
CS25	48341	263.7	35.9	85	1	PV788077	SAMN48543970
CS27	48269	155.9	36	84	2	PV788078	SAMN48543971
CS28	48564	234.8	35.9	86	2	PV788079	SAMN48543972
CS29	47912	168.1	35.9	82	2	PV788080	SAMN48543973
CS30	48027	176.7	36	83	2	PV788081	SAMN48543974
CS31	47932	165.1	36	82	2	PV788082	SAMN48543975
CS32	47700	153.1	35.8	81	1	PV788083	SAMN48543976
CS33	48165	251.8	36	83	2	PV788084	SAMN48543977
CS35	48166	179.7	36	83	2	PV788085	SAMN48543978
CS36	48165	219.4	36	83	2	PV788086	SAMN48543979
CS37	48166	199.1	36	83	2	PV788087	SAMN48543980
CS38	47700	213.6	35.8	81	1	PV788088	SAMN48543981
CS42	47720	38.6	35.8	81	1	PV788089	SAMN48543982
CS43	47700	156.8	35.8	81	1	PV788090	SAMN48543983

## Data Availability

The raw sequence reads were deposited to the NCBI SRA database under BioProject PRJNA1263911. BioSample and GenBank accessions are listed in Table 1 for each phage.
